# Supplementary material for: “It could bring a lot of help to people that aren’t getting help right now”: A qualitative analysis of the impact of virtual care on access to primary care for people with opioid use disorder
Source: PLOS Digit Health. 2026 Mar 17;5(3):e0001299. doi: 10.1371/journal.pdig.0001299 (PMC12994838; doi:10.1371/journal.pdig.0001299)
Supplement: S1 File — (PDF) [file pdig.0001299.s001.pdf]

FP Interview Guide

This study explores changes to primary care access and experiences for people with opioid use disorder resulting from the rapid introduction of virtual care. As a physician, we are interested in hearing about your experiences with and perspectives on providing primary care both in person and virtually for people with opioid use disorder.

As a reminder, this interview will be recorded and transcribed. You will not be identified in any report or presentation; your name will be replaced by a participant code and any identifying information will be obscured. Participation is voluntary. You may end the interview at any time or choose not to answer any specific question. Any questions before we begin? Do you have any questions before we begin?

First, I would like to ask some general background questions about you and your practice.

1. How long have you been practicing as a family physician?

2. Can you tell me a little bit about your training?
   Probes:
   a. Medical school within Canada or international?
   b. Completion of the Provincial Opioid Addiction Treatment Support Program, completion of any other addiction medicine certificate or other post-graduate medical training (e.g., addiction medicine fellowship)?

3. Can you describe your current model of practice?
   Probes:
   a. For example, do you provide care in a community-based practice? ED? Long term care home? Hospital as a hospitalist? Home visits? Split between multiple sites?
   b. Can you broadly describe your patient population/the communities you serve?
   c. How is your practice funded – Fee for Service, Alternative Payment Programmes, Rural Practice Programmes, etc.
   d. Are you a clinic owner?
   e. Do you have any non-physician health professionals working in your practice?

4. Do you belong to any organized networks or physician groups? Which ones?

5. Do you have privileges at any hospitals or other facilities?

6. Would you mind sharing with us the gender you identify with/your pronouns?

Next, I'd like to ask about the population of patients you care for.

7. In which communities do you currently practice and how would you describe them in terms of urban or rural?

8. How many patients with OUD do you see per week (for OUD treatment or other primary care)? What percentage of your patient population has OUD?

9. For approximately how many PWOUD do you feel that you serve as a majority source of their care?

Next, I'd like to ask about your use and experiences with virtual care services.

10. To what extent did you use virtual care within your practice prior to the pandemic?
    Probes:
    a. What percentage of care delivered virtually?
    b. Which modalities (phone visits, video visits, other)?
    c. How did you use virtual care specifically for your patients with OUD compared to your other patients?

11. Can you describe the balance of in-person to virtual care for patients with OUD in your practice currently?
    Probes:
    a. How has that proportion changed over the course of the pandemic/since you first implemented virtual care in your practice?
    b. Which modalities (phone visits, video visits, other)?
    c. Are there any differences in which services you provide in person v. those you provide virtually? To what do you attribute these differences (i.e., comfort, positive/negative experiences, sense of efficacy/appropriateness)

12. What supports/resources have helped you deliver primary care services virtually to patients with OUD?
    Probes:
    a. Role of and comfort with using the requisite technologies (EMR, videoconferencing)?
    b. Introduction of regulatory/billing changes during the pandemic?
    c. Longitudinal relationships with patients?
    d. Guidance documents (for virtual care and/or supporting PWOUD)?
    e. Collaborative care/other health care providers (e.g., clinic nurses, outreach workers/services, community pharmacists)

13. What barriers/challenges have you encountered in starting to offer virtual care services for patients with OUD? What barriers do you expect over the long term?
    Probes:
    a. Patient access to requisite communications technology/confidential spaces?
    b. Changes in patient engagement/frequency of missed or cancelled appointments?
    c. Finding the balance between in person/virtual visits?
    d. Issues with undisclosed/unobserved health concerns/deterioration?

14. How has the introduction of virtual visits changed how your patients are accessing OUD medications and your prescribing workflow? What about the impact of virtual visits on lab testing?
    Probes:
    a. Changes to lab referral process?
    b. Changes to patients' access to/frequency of lab testing?
    c. Patient's duration on OAT (i.e., initiated and/or stabilised prior to COVID-19)
        i. What medications (OAT) do you regularly prescribe for patients and how do they influence remote/virtual care?

15. Can you share some examples where virtual care has worked well in your experiences of providing care to patients with OUD?
    Probe: Have you noticed any ways in which virtual modalities are improving care experiences for patients?

16. Can you share some examples where virtual care has posed a barrier to accessing your patients or has not worked well for in your care of patients with OUD?
    Probes: Have you identified any barriers to care that virtual modalities have introduced for your patients?

17. Overall, what impact has telemedicine had on the quality of your interactions with your patients with OUD?
    Probe:
    a. Better/worse/same quality of interpersonal connection?

18. How have your patients (with opioid use disorder) responded to the switch to telemedicine?
    Probe:
    a. Differences by OAT medication taking, age, length of relationship
    b. Other medications/ongoing primary care management of co-morbidities
    c. What are you doing if patients refuse virtual visits or do not have the ability to attend a virtual visit?

19. When the pandemic is over, do you think you will return to your normal (pre-pandemic) practice patterns or do you think you will offer telemedicine services more often? Why?

20. If you are planning to continue offering remote care, are there any policy or regulatory changes that you would like to see to support your provision of virtual care to PWOUD?
    Probe:
    a. Any (temporary) policies/changes introduced in response to COVID-19 that should be retained/extended?

That's all the questions I have. Is there anything important we have left out of the discussion to inform our understanding of use of virtual care for patients with OUD?

Thank you so much for your time today. As a reminder, we may contact you in the future to verify the accuracy of certain statements once we've transcribed the recording of this interview.
