# Supplementary material for: “It could bring a lot of help to people that aren’t getting help right now”: A qualitative analysis of the impact of virtual care on access to primary care for people with opioid use disorder
Source: PLOS Digit Health. 2026 Mar 17;5(3):e0001299. doi: 10.1371/journal.pdig.0001299 (PMC12994838; doi:10.1371/journal.pdig.0001299)
Supplement: S2 File — (PDF) [file pdig.0001299.s002.pdf]

PWOUD Interview Guide

In this study, we want to gain a better understanding of the impact of virtual models of care on people with opioid use disorder. We are interested in how virtual models impact access to and quality of care for PWOUD, as well as their impact on access to opiate agonist therapies and any treatment for other health conditions.

As a reminder, this interview will be recorded and transcribed. You will not be identified in any report or presentation; your name will be replaced by a participant code and any identifying information will be obscured. Your participation is voluntary. You may end the interview at any time or choose not to answer any specific question. Do you have any questions before we begin?

First, I would like to ask some general background questions.

1. Can you tell me a bit about yourself?
   Probes:
   a. In which community do you live and would you describe it as urban or rural?
   b. How old are you?
   c. Would you mind sharing with us the gender you identify with/your pronouns?
   d. Are you currently on any OAT medications and, if so, which one(s)?
      i. How long have you been on this medication (i.e., were you initiated/stabilised pre-COVID?)
      ii. How often do you currently have to see your physician in-person to receive your OAT meds?
   e. Do you have a regular source of income (formal or informal)?
      i. Does your source of income ever effect your access to primary care/your care provider? (e.g., work hours, access to time off, extended health insurance)
      ii. Do you face any financial barriers in obtaining OAT? (e.g., pharmacy-imposed fees, access to provincial health insurance/coverage)
   f. Has your race, ethnicity, Indigeneity, culture, or preferred language every impacted your experiences with or access to primary care/treatment for opioid use disorder?
   g. Do you have regular/consistent access to a phone (your own number) or a computer with the internet?

Next, I'd like to get a sense of your routine interactions with health services

2. To start, can you describe for me (as much as you are comfortable) any routine interactions you have with health services?

Note: If appropriate, take this opportunity to distinguish between interactions with primary care and other (e.g., acute) health care needs with the participant

3. Is there one doctor's group, health centre, or clinic you usually go to for most of your medical care?
   a. *If yes, ask:* At that location, is there one doctor, nurse, or health care provider that you normally see?
      Probe

        i. Approximately how long have you been going to this place/seeing this individual (physician/nurse/provider) for your medical care?
    b. *If no, ask:* When you need to receive non-urgent medical care, where do you go?
    Probe
        i. Walk-in clinic, urgent care, harm reduction service, community organisation, hospital/emergency department

4. In an average month, how frequently would you estimate that you seek professional medical care or consult with a family doctor?

5. What methods do you normally use to consult with physicians (e.g., in person/virtual visit)
    a. (If in-person) How far do you have to travel to visit [a/your family doctor/clinic]? Do you have access to a car or public transportation?
    b. (If virtual) Telephone/video consultation?

In the next set of questions, I'd like to discuss your health service experiences during the COVID19 pandemic. As you may recall, in March 2020, the government of BC introduced a stay-at-home closure. During that time, health services were instructed to minimise their in-person visits as much as possible and virtual care was widely adopted. Since that initial closure, most doctors have welcomed patients back for in-person visits to varying degrees, but virtual care has also continued.

6. Have you [seen your family doctor/sought primary care] since the pandemic was declared?
    Probes:
    a. How frequently?
    b. How? (in person, virtually)

7. Can you tell me about any changes in accessing your family doctor/regular primary care (clinic) that you've encountered during the pandemic?
    Probes:
    a. Positive changes? Negative changes?
    b. Any impacts on health/wellbeing?
    c. What role has virtual care played in this?

8. Can you tell me about any experience(s) during the past two years when you had an appointment with your doctor that did not happen in-person?
    Probes:
    a. Was it by phone/video?
    b. How frequently?
    c. Only virtual, or interspersed with in-person appointments?
    d. Scheduled appointment or ad hoc?
    e. What did you like/not like about your virtual appointment?

9. Has there ever been a time during the pandemic where your physician wanted to meet with you by phone or video, but you wanted to meet in-person or you didn't have the capacity for a virtual visit (for example, if you couldn't access a phone/computer/internet/mobile data)?
    Probes:
    a. Alternatively, have you ever had issues accessing your family physicians because no virtual care option was provided?

   b. Can you share with me how that was resolved? (e.g., appointment cancelled/no-show, alternative arrangements made)
   c. Can you tell me how that made you feel?

[For participants who have experiences with virtual primary care]
Next, I'd like to focus on your experiences with telephone/video consultations with your family doctor/primary care provider – for short, I'll refer to these as virtual interactions.

10. When you think about your virtual interactions with your doctor, how do those experiences compare to in-person visits you've had with the same physician/clinic?
    Probes:
    a. Better/worse/same quality of interpersonal connection?
    b. Length of visit?

11. Do you feel that you are able to fully address your health care needs during a virtual interaction?
    Probes:
    a. Ability to address multiple issues in a single visit?
    b. Any differences in efficacy of virtual visit to address different health concerns?
    c. Are there any health issues that you aren't comfortable discussing through a virtual interaction or that you might not mention to a physician if they didn't see/ask about?

12. Are there any resources or supports that have influenced your experiences with virtual care?

13. Once the pandemic is over and normal health care services resume, what role do you think virtual services should play in (your) primary care?
    Probe
    a. How would you feel about a remote viewing system to witness your daily dose of methadone/suboxone – would that be preferrable to a daily witness by a pharmacist?

[For participants who have not experienced virtual primary care]
Next, I'd like to hear your thoughts on telephone/video consultations with family doctors/primary care providers. You've already mentioned that you haven't had any experiences with these, but I'm interested in hearing any thoughts you may have about this option.

14. How do you feel about the idea of accessing primary care/a family physician virtually?
    Probes:
    a. If interested/positive, preference for phone/video?
    b. How frequently would you want to consult with health services virtually v. in person?
    c. Why do you think this would be something you'd like?
    d. How might it improve your health/wellbeing?
    e. How does the pandemic inform how you feel about the idea of virtual care? (e.g., outside of a pandemic, would you still be interested in accessing care virtually?)
    f. If there were a remote viewing system to witness your daily dose of methadone/suboxone, would you prefer that over the daily witness by a pharmacist?

15. What challenges, if any, might you face if your regular care provider/primary care clinic were to introduce virtual consultations to their practice?

Probes:
a. Technologies (phone, phone number, computer, internet)?
b. Longitudinal relationship with a family physician/primary care clinic team?
c. Private/secure space?

16. What supports would you need to be able to (comfortably) consult with your doctor virtually?
Probes:
a. Technologies (phone, phone number, computer, internet)?
b. Longitudinal relationship with a family physician/primary care clinic team?
c. Private/secure space?

That's all the questions I have today. Is there anything important we have left out of this discussion to inform our understanding of virtual care for individuals with an opioid use disorder?

Thank you so much for your time today. As a reminder, we may contact you in the future to verify the accuracy of certain statements once we've transcribed the recording of this interview.
