# Supplementary material for: “It could bring a lot of help to people that aren’t getting help right now”: A qualitative analysis of the impact of virtual care on access to primary care for people with opioid use disorder
Source: PLOS Digit Health. 2026 Mar 17;5(3):e0001299. doi: 10.1371/journal.pdig.0001299 (PMC12994838; doi:10.1371/journal.pdig.0001299)
Supplement: S3 File — (PDF) [file pdig.0001299.s003.pdf]

Code Label		Code Definition
Participant characteristics & care context		
1	Participant background	
	1a FP	FP training/background/experience, including background of providing care to PWOUD and completion of any addiction medicine/OAT prescribing programmes
	1b PWOUD	Participants lived/living experience with OUD, personal history (age, location, current profession/income etc); including description of any ongoing health conditions/needs
2	OAT/SS Rx	Current OAT/safer supply meds prescribing (FP) or receiving (PWOUD); duration of prescription/current regimen for latter
3	Care context	
	3a Patient characteristics	Description of patients seen/served in a given clinic/care setting, including number of pts with PWOUD to whom FP provide care/for whom FP serve as majority source of care
	3b Staff complement	Descriptions of staff who FPs work with in their different clinics/care settings/staff that PWOUDs interact with during appointments/health care contexts
	3c Practice model	Description of the practice model(s) in which FPs work/patients access care; includes whether/not FP identifies as a clinic owner, including descriptions of processes/workflows that are specific to a particular clinic/practice model
	3d Payment model	Description of the funding model(s) by which FPs are remunerated
	3e Community setting	Description of community/geographic setting in which participants provide/receive care
	3f Scope of care	Description of care available to PWOUD and/or provided by FP (i.e., primary care, OAT, safer supply) in a given care context/across different care contexts
	3g Community services	Descriptions of services and (non-primary) care provision in a given community/setting
4	Access to primary care	Expressions of patient access to (longitudinal) primary care, including patient choice in providers (e.g. where/from whom do patients access primary care)
	4a Frequency of health system interactions	Descriptions of regular interactions of health services; how often they engage with the health system (may include descriptions of regular interactions outside of primary care)

5	Technology	Description of technologies to which participants have regular access; PWOUD – personal v. public access; FP access in practice settings
6	Stability	Descriptions of patient stability or lack thereof (living situation, OAT management); stability as an influencing factor in patient/provider care decisions
**Care experiences**		
7	Pre-pandemic	Descriptions of care experiences (provided/received) prior to COVID-19
8	Pandemic	Descriptions of care experiences (provided/received) during the COVID-10 pandemic (ie, during pandemic public health precautions)
9	Post-pandemic	Descriptions of care experiences (provided/received) after pandemic precautions were lifted
10	Modality of care	Patient/provider preferences and decision-making regarding modality (in person v. virtual); discussion of challenges/benefits associated with different modalities; can include discussions of ambivalence between in person/virtual and comparisons between the two
	10a In-person care	Distinct descriptions of in-person care experiences
	10b Virtual care	Distinct descriptions of virtual care experiences
11	Virtual care platform	Patient/provider preferences, decision-making regarding platform (phone v. video); challenges/benefits associated with different platforms
	11a Security concerns	Any descriptions (+/-) of security features/concerns associated with different virtual interactions/interfaces/modalities
12	Preferred care	What patients/physicians would like their provision/receipt of care to be (going forward), including any supports/resources needed to facilitate this; may include descriptions of current care provision if they do not wish this to change
**Features associated with care (+/–)**		
13	Advocacy	Descriptions of patients speaking up for themselves/the care they want/need (ie, advocating for desired/needed care); can include FP discussing patients' self-advocacy or FPs advocating for their patients
14	Attendance	Any discussion of (primary care) appointment attendance; improvements/changes to; factors that may influence patient attendance rates
15	Autonomy	Descriptions of desires for/experiences of self-determination (as a patient, for patients) in care experiences; includes discussion of agency

16	Comfort	Comfort/discomfort with care experiences, both providing/receiving, including changes in comfort level; differences in comfort based on patient/provider/specific OAT meds
17	Confidentiality	Any issues (or non-issues) with confidentiality during provision/receipt of care, including descriptions of the ways in which confidentiality can be assessed/achieved
18	Convenience	Descriptions of any (in)convenience associated with care experiences - FP providing/PWOUD receiving
19	OUD care	Descriptions of OUD-specific care/OAT management by providers/patients, including urine drug screen requirements (frequency) and discussions related to OAT med diversion; includes discussion of broader addictions care that is not specific to OAT as well as access to/provision of safer supply and its associated processes
20	Patient centred care	Ways in which providers work to provide patient centred care, PWOUD desires/needs to achieve patient centred care; descriptions of patient centred care (to be used when patient centred care is expressly stated)
21	Preparation	Descriptions of patient/provider preparation (or lack there of) for appointments
22	Policy/regulation	Policies, rules, regulations, requirements that influence physicians' provision of care, whether those are set by a clinic/health authority, college, or province; distinct from clinical guidelines that provide suggestions or inform how they *should* provide care; includes PWOUD experiences of policies & regulations
23	Primary care	descriptions of care provision/experiences covering broader health care needs of PWOUD/patients (chronic illness management, mental health care) (e.g., what does it look like/involve when you provide (FP)/receive (PWOUD) primary care)
24	Quality of care	Patient/provider assessments of the quality of care they receive/are able to provide; includes quality of interpersonal connection, interaction, patient/provider engagement during appointments as well as anecdotes of quality of care and health outcomes
25	Resources (+/-)	Factors or tools that influence the provision/receipt of care (eg. technology, collateral, guidelines, RACE line etc) - available or desired; includes description of resources (or the absence of resources) that negatively influence care
2	25a Remuneration	Specific reference to ways in which FP payment/billings influence provision of care to PWOUD
	25b HCPs (Ext)	Ppl working formally within the health system that support provision/receipt of care to PWOUD - outside of a given care team
	25c HCPs (Int)	Ppl working formally within the health system that support provision/receipt of care to PWOUD - within the same care team
	25d Community services/staff	Services and staff, outside of formal health system, that support provision of/access to care for PWOUD

26	Duration	Length (time) of primary care appointments and any associated implications (+/-)
27	Stigma/Discrimination	Experiences or descriptions of discrimination based on participant identity/social experiences (e.g., racism, OUD-related, income/housing)
28	Timeliness	Descriptions of the length of time it takes to access a primary care provider/clinic; consequences associated with wait times
29	Therapeutic relationship	Ways in which longitudinal/therapeutic relationships support/hinder provisions of care to/by PWOUD, including discussion of trust between patients and clinic/clinic staff (including providers)
30	Unintended consequences	Surprising/unanticipated ways that changes in primary care have impacted provider/patient interactions (+/-)
Other		
31	Pandemic impact	Ways in which the pandemic has affected primary/health care experiences, practice settings, care provision, access to care, and health outcomes (beyond changes to virtual care)
32	Remote viewing system	Participant responses to question about preference/potential of a remote viewing system for OAT
