# Supplementary material for: “It could bring a lot of help to people that aren’t getting help right now”: A qualitative analysis of the impact of virtual care on access to primary care for people with opioid use disorder
Source: PLOS Digit Health. 2026 Mar 17;5(3):e0001299. doi: 10.1371/journal.pdig.0001299 (PMC12994838; doi:10.1371/journal.pdig.0001299)
Supplement: Attachment — Submitted filename: VPC OUD_Access R&R Letter (PLOS DH).pdf [file pdig.0001299.s005.pdf]

THE UNIVERSITY OF BRITISH COLUMBIA

Faculty of Medicine
The University of British Columbia
317-2194 Health Sciences Mall
Vancouver, BC V6T 1Z3

Phone 604 822 2421
Fax 604 822 6061
www.med.ubc.ca

Girum Tareke Zewude, PhD
Academic Editor
PLOS Digital Health

February 10, 2026

Dear Dr. Zewude,

Thank you for reviewing the manuscript entitled, *“It could bring a lot of help to people that aren't getting help right now”: A qualitative analysis of the impact of virtual care on access to primary care for people with opioid use disorder.* We have revised the manuscript in track changes to address the reviewers’ comments, listed below with our responses. Any in-text changes are accompanied by the corresponding line numbers from the manuscript with track changes.

---

1. **Reviewer 1:** The authors have provided a look at how persons with opioid use disorder might (or do) benefit from virtual care options. This is an interesting work and contributes to the knowledge building around virtual care in underserved populations. I suggest some minor revisions, and in particular emphasising the discussion around virtual care in the first section of the results section (accessibility).

1.1. Check spelling/grammar throughout paper - for example on line 84, 'barriers' is incorrectly capitalised, and British Columbia is used in both its expanded and abbreviated state throughout.
Thank you for bringing these errors to our attention. We have corrected the capitalization of ‘barriers’ (line 83) and the inconsistent use of BC and British Columbia throughout the manuscript. Upon completing all revisions, we thoroughly reread our manuscript to ensure accurate spelling and grammar throughout.

1.2. In the methods section, it is mentioned that recruitment was pursued until sufficient data was collected. Perhaps you could expand on how you made this determination. You have supporting citations for the statement but I would like to know how the methods from these articles translated into how you actually determined you had sufficient data.
We have added a sentence (line 135-137) to clarify how we assessed the sufficiency of our data: *We assessed sufficiency of FP and PWOUD data independently based on a combination of the amount of data and variation of experiences within the data, including disconfirming and discrepant cases [44,46].*

1.3. In the data collection and analysis section, the lengths of the interviews (range and average) seem to be more results than methods. Consider moving this data to the results section.
Thank you for this suggestion; we have moved the interview length from the Methods to the Results section (line 202-203).

1.4. It would be helpful to have your codebook as supplementary material if possible.

We are pleased to provide our codebook as supplementary material (Supplementary File 3) and have added a reference to this in-text (line 159).

1.5.

1.5.1. For the first part of the accessibility section in the results, it is unclear how the first quote relates to virtual care. I'm assuming this doctor then sees those remote patients virtually, and this helps them keep these patients on the attachment list when they struggle to find care in their location, but this doesn't come through very clearly.

We have added additional data from this participant (line 218-219) to better situate the quote: *"...patients who move away, virtual care allows them to continually access me from a big distance…"*

1.5.2. Similarly with the last quote of the section (about the patient with MS), you briefly mention the advantage of virtual care for this patient, but it doesn't really come through clearly from the patient's quote or your writing. Perhaps be a bit more explicit with what the advantages are here.

We have restructured the framing of this quote to enhance clarity (line 248-250; line 256-258), and provided additional data to support the framing (line 257-258): *("I [wouldn't] have to travel" [PWOUD10])*

1.5.3. Finally, at the end of this section you say "the concept of a virtual appointment suggested to this participant that their health might improve" - this statement is not clear to me - is this something that you as the interviewer suggested to the patient or something that the patient came up with when learning about virtual care options?

We have rephrased this sentence to include the prompt we explored in the interview and have provided the participant's response as a parenthetical (line 259-260): *("I would talk to my doctor a lot more" [PWOUD10])*

1.6. The quote and its supporting information would benefit from a bit more discussion. Since the paper up to this point has mostly focused on positive aspects of virtual care, this aspect of requiring in-person visits in some cases despite the virtual offering could have more detail.

We appreciate the reviewer's prompt to further explore this quote and have added additional discussion (line 297-301), focusing on the limited availability of video-based virtual care amongst our participants and the value that both FPs and PWOUD placed on visual cues: *A key aspect of this continued reliance on in-person appointments was the value of FPs being able to use their full range of diagnostic tools, particularly 'laying eyes on' individuals and exploring issues that they may not identify without visual cues. This is likely related to the dominance of phone-based virtual appointments, as most video-based appointments have been abandoned due to inconvenience and the digital divide – both perceived and actual.*

2. **Reviewer 2:** I thought this paper overall was in decent shape even in its current form. It would benefit from an edit pass and some minor revisions.

2.1. Have other OUD studies used this same theory?

We are unaware of any previous OUD studies that have utilized Saurman's updated theory of access, however several substance and opioid use disorder studies have referred to the original conceptualization of access by Penchansky and Thomas in their analyses (see, for example: 10.1186/s13063-025-09364-x; 10.1186/s13722-025-00592-9; 10.1177/00220426251394665; 10.1097/ADM.0000000000001602). Both Penchansky and Thomas' original and Saurman's updated frameworks are frequently employed by researchers in studies on access to primary care and other essential health services for socially and clinically complex populations. This framework has been highlighted in such research for its utility in exploring patients' and/or (health service) consumers' perspectives of access, as we do in this analysis (see, for example: 10.1097/ADM.0000000000001602 or 10.17645/si.10418).

2.2. Do other studies on virtual care delivery have similar findings to OUD?

We have added a sentence in our Introduction to summarize key findings on virtual care, specifically focusing on how it impacts access to primary care (line 100-103): *The broader body of research indicates that virtual modalities can support improved access to primary care for some patients, but several factors (e.g., the digital divide, health literacy, patient demographics and social location) may impede equity of access to virtual care offerings [27–29].*

2.3. Pandemic is mentioned here although does not show up anywhere in the abstract

In revising the abstract to improve its narrative flow (per Reviewer 3's comment below), we have added a reference to the COVID-19 pandemic (line 25).

2.4. Barriers capitalized

Thank you for bringing this error to our attention; we have corrected the capitalization of 'Barriers' (line 83).

2.5. Is this typical to group residents with practising physicians?

We decided to include medical residents in our family physician eligibility criteria – if they responded to our recruitment efforts – as we believed their experiences with PWOUD and virtual modalities would be equally valuable to capture and explore. No medical residents ultimately responded during recruitment.

2.6. Is the development of interview guides documented?

We appreciate Reviewer 2's attentiveness to appropriate representation and documentation of the creation of data collection tools and would direct them to our Ethics and Study Rigour subsection of the Methods,

where we originally noted (line 191-192): "We prepared interview guides in consultation with health system and substance use researchers, family physicians, and PWOUD."

2.7. Did all interviewers hold a relevant degree in health sciences?

While all our interviewers hold a variety of graduate level degrees - in health and social sciences – we do not consider this to be a *de facto* indicator of skilled interviewers. Instead, we assessed interviewing qualification based on years of prior experience conducting interviews with a variety of participant populations, understandings of the local primary care context and opioid use disorder, and competencies in cultural awareness, humility, and safety.

2.8. Can you back up the interview findings with any more journal references?

We appreciate Reviewer 2's request to provide more references to support our findings. Accordingly, we have added additional references throughout our Discussion (lines 483, 492, 522, 525, 527).

---

3. Reviewer 3: This paper seeks to understand the virtual care experiences of family physicians and people with opioid use disorders, relying on a thematic analysis of semi-structured interviews with both participant groups. Overall, the paper is well-written, methodologically rigorous and sound, and should be of interest to the research community. I have a few small nitpicks that should be easy to address.

3.1. While the majority of the paper is well-written, the abstract is a bit terse in its writing style. The authors should consider some light edits to improve the flow here.

We appreciate this suggestion from Reviewer 3 and have revisited the abstract to improve its narrative flow (line 25-43).

3.2. Were participants compensated for their participation in the study?

Yes, both FP and PWOUD participants were provided honoraria in recognition of their time for participating in an interview. We have opted not to include information about participant honoraria in this manuscript as we have detailed our approach to interview honorariums previously in our published study protocol, which is cited at the outset of the Methods section (line 116).

3.3. It would be nice to include some more details on which or at least how many authors conducted the interviews and analysis. Was it all authors, a subset, or perhaps even researchers not listed as authors included (I expect this isn't the case, as I would expect this to be in the acknowledgements at least if it were the case)?

We have clarified the which authors were involved at various stages of data collection and coding by adding the initials of interviewers (line 139), codebook developers (line 153), and the final group of coders (line 159-160).

3.4. Barriers is capitalized

Thank you for bringing this to our attention; we have corrected the capitalization of ‘Barriers’ (line 83).

3.5. While the Strengths and Limitations section does mention the fact that participants were recruited solely from British Columbia, it’s not really framed as a limitation. The fact that this might limit the applicability of findings to other geographic locations should be more clearly stated.

We agree with Reviewer 3 that our previous discussion of the study’s limitations with respect to geography was not clearly articulated. We have added to our recognition of the limits to our findings generalizability (line 538-540): *… these findings may not be generalizable to other populations in BC and those in other jurisdictions with different regulatory environments that inform health service delivery and access.*

3.6. From skimming the references, I don’t see any references from the CHI/CSCW literature. I don’t work in this specific area (i.e., opioid use) so I’m not sure if there is work specifically on this, but there are certainly researchers doing qualitative work in similar spaces (i.e. healthcare access), so there might be. If not already done, it might be worth doing a quick search to see if there are relevant works to cite from those communities.

We are grateful to Reviewer 3 for introducing us to the CHI/CSCW body of work. After careful review, we did not find any literature addressing OUD specifically within this subject area. Literature on CHI/CSCW in primary care, however, may complement other analyses of this data that are ongoing (e.g., quality of virtual primary care experiences).

We hope that we have satisfactorily addressed the concerns raised by the reviewers. We appreciate their detailed, constructive feedback and feel that the manuscript is stronger as a result.

On behalf of all authors,

Dr. Rita K. McCracken, MD, PhD, CCFP (COE), FCFP
Clinical Faculty, Department of Family Practice, University of British Columbia
rita.mccracken@ubc.ca
